# In vitro inactivation of SARS-CoV-2 by commonly used disinfection products and methods

**DOI:** 10.1038/s41598-021-82148-w

**Published:** 2021-01-28

**Authors:** Guo Xiling, Chen Yin, Wang Ling, Wu Xiaosong, Fan Jingjing, Li Fang, Zeng Xiaoyan, Ge Yiyue, Chi Ying, Cui Lunbiao, Zhang Liubo, Sun Hong, Xu Yan

**Affiliations:** 1 Jiangsu Provincial Center for Disease Prevention and Control, 172 Jiangsu Road, Nanjing, 210009 Jiangsu China; 2 Key Laboratory of Enteric Pathogenic Microbiology, Ministry Health, Institute of Pathogenic Microbiology, 172 Jiangsu Road, Nanjing, 210009 China; 3 National Institute of Environmental Health, Chinese Center for Disease Control and Prevention, Beijing, 100021 China

**Keywords:** SARS-CoV-2, Pathogens

## Abstract

Severe acute respiratory syndrome coronavirus-2 (SARS-CoV-2) infection is currently a global pandemic, and there are limited laboratory studies targeting pathogen resistance. This study aimed to investigate the effect of selected disinfection products and methods on the inactivation of SARS-CoV-2 in the laboratory. We used quantitative suspension testing to evaluate the effectiveness of the disinfectant/method. Available chlorine of 250 mg/L, 500 mg/L, and 1000 mg/L required 20 min, 5 min, and 0.5 min to inactivate SARS-CoV-2, respectively. A 600-fold dilution of 17% concentration of di-N-decyl dimethyl ammonium bromide (283 mg/L) and the same concentration of di-N-decyl dimethyl ammonium chloride required only 0.5 min to inactivate the virus efficiently. At 30% concentration for 1 min and 40% and above for 0.5 min, ethanol could efficiently inactivate SARS-CoV-2. Heat takes approximately 30 min at 56 °C, 10 min above 70 °C, or 5 min above 90 °C to inactivate the virus. The chlorinated disinfectants, Di-N-decyl dimethyl ammonium bromide/chloride, ethanol, and heat could effectively inactivate SARS-CoV-2 in the laboratory test. The response of SARS-CoV-2 to disinfectants is very similar to that of SARS-CoV.

## Introduction

Severe acute respiratory syndrome-coronavirus-2 (SARS-CoV-2)^1^ is a novel virus that is first reported in Wuhan, China, and it is responsible for causing coronavirus disease 2019 (COVID-19)^2^. The World Health Organization (WHO) declared COVID-19 a pandemic, and by January 7, 2021, there were over 85 million cases and 1.8 million deaths reported throughout 222 countries and territories worldwide^3^. On January 12, 2020, SARS-CoV-2 was first isolated and identified by the Chinese Center for Disease Control and Prevention^4^. SARS-CoV-2 is the seventh human coronavirus isolated after the coronavirus strains 229E, OC43, NL63, and HKU1, and the more pathogenic human coronaviruses discovered in recent years, namely the severe acute respiratory syndrome coronavirus (SARS-CoV) in 2002–2003 and the Middle East respiratory syndrome coronavirus (MERS-CoV) in 2012. SARS-CoV-2 is a coronavirus belonging to the genus of beta coronavirus, which is enveloped with round or oval particles, often polymorphic, and having a diameter ranging from 60 to 140 nm^5^. The SARS-CoV-2 gene profile significantly differs from that of SARS-CoV and MERS-CoV^4^.

SARS-CoV-2 is highly transmissible from person to person, and it is a larger threat to human beings than previous coronavirus strains as it has a much higher reproduction number (R_0_ = 2.2)^6^, implying that the number of cases generated by one infected person is higher than previous outbreaks. The persistence time of SARS-CoV-2 on inanimate surfaces varies from a few minutes up to 1 month, depending on the environmental conditions^7^. Although the vaccines are already available^8,9^, there are still significant challenges to achieving global population immunization, including vaccine storage, transportation, and availability. Similar to SARS, no treatment is currently available to treat COVID-19. Various approaches have been used to treat this COVID-19 during this pandemic, but none is targeted or entirely effective. The treatments now being administered are essentially empirical and symptomatic and dependent on the illness’s severity^10,11^. Therefore, in the absence of enough vaccines and effective drugs, disinfection plays a vital role in preventing and controlling the spread of COVID-19. Like SARS, in a significant number of COVID-19 cases in China, epidemiological investigations could not find evidence of direct close contact with other confirmed patients; therefore, at least some of these cases may be caused by indirect transmission. Although transmission was believed to be mainly achieved by direct physical contact with an infected patient or by respiratory droplets, several well-described clusters of infection were difficult to explain by these routes^12^. Therefore, a crucial preventive measure against COVID-19 is the sufficient inactivation of SARS-CoV-2 using available disinfection methods.

Our current knowledge of SARS-CoV-2 disinfection is based on previous coronavirus studies. Coronaviruses are lipophilic viruses surrounded by an envelope, which makes them easy to inactivate. Theoretically, all approved commercial disinfectants, disinfection devices, and physical disinfection methods effectively inactivate SARS-CoV-2. However, laboratory studies on SARS-CoV-2 remain very limited^2^. The WHO-recommended hand scrub formulations and alcohol disinfectants are based upon research that used quantitative suspension tests to confirm the effectiveness of these approaches^13^. However, studies on heat disinfection and some other chemical disinfectants, especially weak disinfectants, have been less frequently evaluated. Currently, chlorinated disinfectants are the preferred disinfection products in Chinese practice, WHO’s guidance^12^, or the United States Environmental Protection Agency (US EPA)^14^. This is a prudent approach, and there has always been a concern about over-sanitizing, including excessive disinfection treatments and relevant subjects. Radical practices, in particular, use chlorinated disinfectants to treat car wheels and even the human body. Chlorinated disinfectants are also unsuitable for the disinfection of high-grade vehicles, such as aircraft and high-speed trains, whose components may corrode. Ethanol disinfectants are WHO-recommended hand disinfection products with a wide range of applications^13^, but testing is still needed to understand the required minimum concentration to inactivate SARS-CoV-2 and the necessary minimum time required. There is also some doubt about the effect of heat disinfection on SARS-CoV-2, as previous studies on SARS-CoV have given different results on whether 56 °C for 30 min can inactivate SARS-CoV^10,15^. Besides, there is no experimental data to support the time required and inactivation effect of quaternary ammonium compounds (QAC) disinfectants on SARS-CoV-2, although QAC is listed on the US EPA product list.

Given this background, we used a sequenced SARS-CoV-2 strain from an infected person in Jiangsu, China, to evaluate the inactivation effect of commonly used disinfection methods on SARS-CoV-2. We tested the effect of chlorine-containing disinfectants, two kinds of QAC named Di-N-decyl dimethyl ammonium bromide (DNB) and Di-N-decyl dimethyl ammonium chloride (DNC), ethanol, and heat on SARS-CoV-2 inactivation, to provide important information on their efficiency on virus inactivation.

## Material and methods

We conducted this study in the Biosafety Level III (BSL-3) laboratory of the Center for Disease Control and Prevention in Jiangsu Province (Jiangsu CDC), accredited by the ministry of science and technology of China. Researchers in the BSL-3 laboratory was allowed to conduct experiments using SARS-CoV-2, including virus isolation. The specific technical methods for disinfection testing are based on the Technical Specification for the Inspection of Disinfection Products^16^. This study was exempted from ethical review by the Ethics Committee of the Jiangsu CDC because the study did not use human-related biological samples directly.

### Cell culture

Vero-E6 cells were acquired from the Cell Bank of the Chinese Academy of Sciences Typical Culture Collection Committee. Cells were cultured using Dulbecco’s Modified Eagle Medium (DMEM) supplemented with 10% fetal bovine serum (FBS), 100 μg/mL of streptomycin, and 100 IU/mL of penicillin (Gibco; ThermoFisher). The cells were incubated in a 37 °C, 5% CO_2_ incubator. Cell growth was observed daily, and disinfection tests were performed once the cells had covered a single layer.

### Preparation of virus suspensions

We thawed the frozen SARS-CoV-2 strain (SARS-CoV-2/human/CHN/Changzhou_JS27/2020, GENEBANK NO. MT534630) in a 37 °C water bath. The virus was then diluted tenfold with cell maintenance solution (DMEM + 2% FBS) and inoculated in cell vials that were covered with a monolayer cell. The culture flask was then placed in a 37 °C incubator to allow the virus to be adsorbed and grow with the cells. We observed the cellular lesions daily and harvested the virus when three-fourths of the cells showed lesions. We crushed host cells with ultrasound under ice-bath conditions to release the virus. Then, samples were centrifuged as soon as possible (500 g, 15 min) to remove sediment (mainly cellular debris), and the supernatant containing the viral suspension was collected. The viral suspension was divided into sterile centrifuge tubes (1.5 mL) at 1.0 mL per tube and stored at − 80 °C. We took one viral suspension and measured its viral titer. Viral titers were calculated by expressing them as the median tissue culture infective dose (TCID_50_). The detailed methodology was based on Chinese disinfection specifications^16^. The TCID_50_ for viral titers in this test was 10^−5.5^/0.1 mL.

### Tests to determine the neutralizing agent

A neutralizing agent is used to terminate the disinfectant’s action and thus accurately control the disinfection time. Therefore, we need to select the appropriate neutralizing agent for each disinfectant before evaluating the disinfection effect. Neutralizing agents need to have a proper disabling effect on the test disinfectant and must not have harmful or adverse effects on the viruses and cell lines used in the test. Tests were conducted as per the Chinese disinfection specifications^16^. In brief, each chemical disinfectant was used at two different concentrations to test two groups of neutralizing agents’ efficacy. The specific experimental design is shown in Table 1.

**Table 1:** Chemical information and neutralizer experiments.

Disinfectant	Active ingredient	Test I		Test II	
		Dose	Neutralizer	Dose	Neutralizer
Chlorine disinfectant	Trichloroisocyanuric acid	1000 mg/L^a^	1 g/L sodium thiosulfate	500 mg/L^a^	0.5 g/L sodium thiosulfate
Quaternary ammonium salt	Di-N-decyldimethylammonium bromide/chloride	850 mg/L	0.5 g/L sodium thiosulfate + 0.5 g/L lecithin	170 mg/L	0.1 g/L sodium thiosulfate + 0.1 g/L lecithin
Ethanol	Ethanol, 99.7%	75%	DMEM^b^ solution	20%	DMEM^b^ solution

^a^Concentrations of available chlorine.

^b^Dulbecco’s Modified Eagle Medium.

Overall, we used suspension quantification and designed six sets of tests for each neutralizer to characterize the neutralization efficacy. Group 1: Disinfectant + virus suspension; Group 2: (Disinfectant + virus suspension) + neutralizer; Group 3: Neutralizer + virus suspension; Group 4: (Disinfectant + neutralizer) + virus suspension; Group 5: Virus suspensions; Group 6: Cellular controls.

We performed this by placing 0.4 mL of 1.25 × test concentration disinfectant solution (or neutralizer) in a test tube, placing it in a 20 °C ± 1 °C water bath for 5 min, adding 0.1 mL of virus suspension, and mixing well. The mixture was allowed to react for a predetermined time before 0.1 mL of the mixture was taken and mixed with 0.9 mL of the neutralizer (or deionized water). The final sample was aspirated (or tenfold serially diluted with DMEM), and the subsequent virus titer was determined.

### Quantitative suspension tests

**Figure 1:**
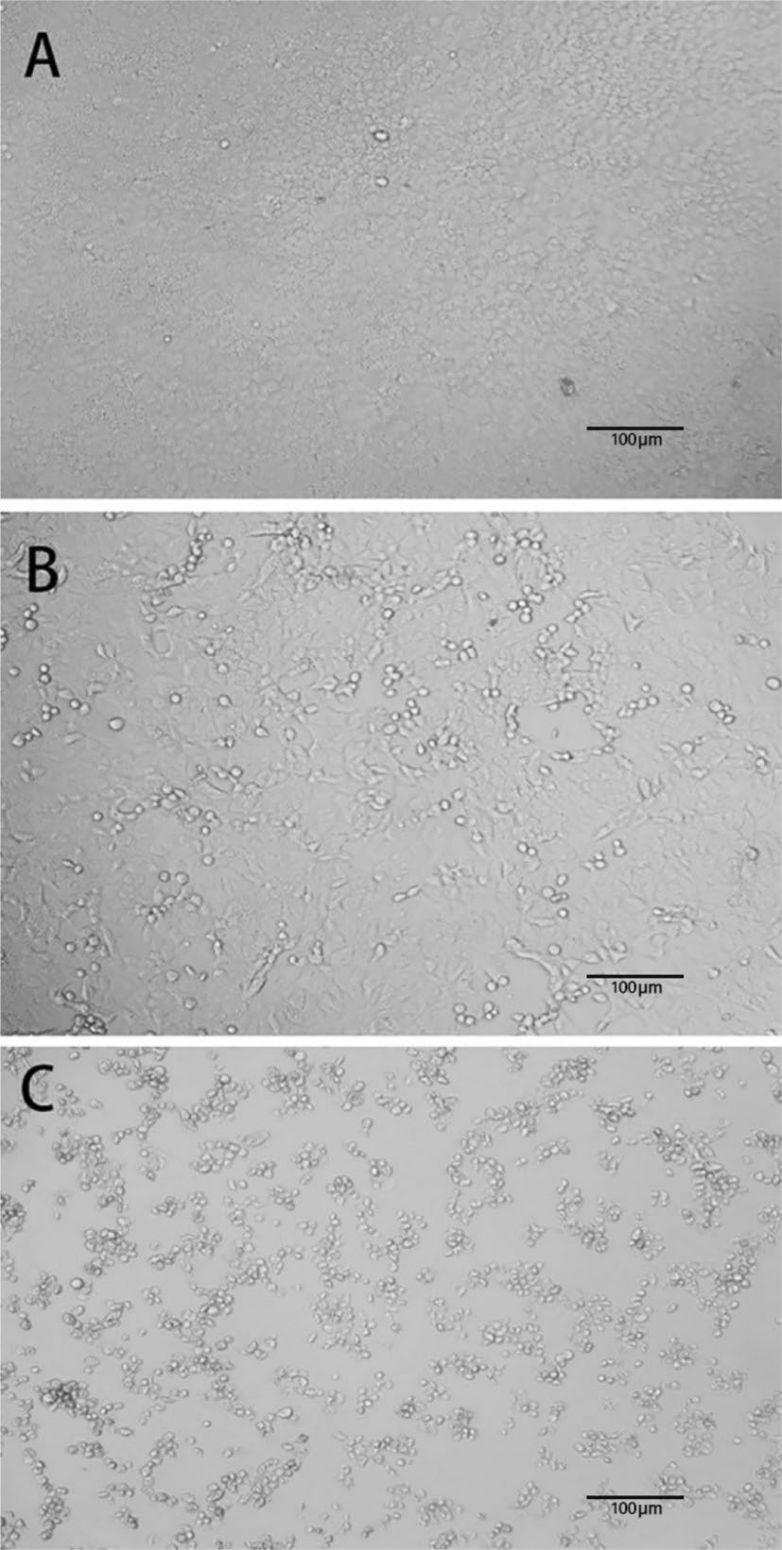
Morphology of Vero-E6 cells under a tenfold magnification in different infection states. (**A**) Intact cells. Vero-E6 cells formed a complete monolayer before SARS-CoV-2 infection. (**B**) Partially infected cells. After SARS-CoV-2 and disinfectant (20% ethanol) addition, some cells died and dislodged from the bottom of the 96-well plate, indicating incomplete disinfectant action. (**C**) Fully infected cells. After infection with SARS-CoV-2 alone, all cells died and were completely detached from the bottom of the 96-well plate.

We used quantitative suspension tests to evaluate the inactivation effect of chemical disinfectants on SARC-CoV-2. The test disinfectants were prepared in sterile standard hard water to an aqueous solution of 1.25 times the test concentration. The viral suspension was mixed with 3% bovine serum albumin (BSA) solution in a 1 × (1:1) mixture and placed in a water bath at 20 °C for 5 min. In each sterile test tube, 0.2 mL of viral suspension was immediately mixed with 0.8 mL of disinfectant and allowed to react for a specified time. Then, 0.1 mL of the reaction solution was added to a small test tube containing 0.9 mL of the neutralizer solution and mixed for 10 min, before undergoing a tenfold series dilution with cell culture solution. Samples were seeded onto 96-well cell culture plates (1 × 10^4^ cells/well) with cells growing into monolayers and four wells per titration. The 96-well cell culture plate was incubated at 37 °C in a 5% CO_2_ incubator for 1–2 h, and the cell culture solution was replaced and continued for 5 days. The results were observed daily (Fig. 1). Both positive and negative controls were performed for each test. The positive control group used sterile deionized water instead of disinfectant, and the negative control group used only cell maintenance solution. Viral titers were determined for each group, and TCID_50_ and log inactivation values were calculated. The test was repeated at least three times.

### Heat inactivation methods

After the temperature of the incubator reached the desired temperature, a sealed Eppendorf tube containing 0.3 mL of SARS-CoV-2 viral fluid (10^−5.5^/0.1 mL for TCID_50_) was inserted into the incubator wells, and the Eppendorf tube was placed in an ice-water bath immediately after the set heating time. Vero-E6 cells were inoculated with the virus, alongside inactivated viral controls and normal cell controls. The cells were incubated for 5 days for observation; the occurrence of lesions was recorded.

### Statistics and analysis

The average killing log-value was a measure of the effectiveness of the disinfectant in inactivating the virus. This value was calculated by the following formula:

$${\text{Average killing log-value }} = {\hbox{ log }}{{\text{N}}_0} - {\text{log Nx}}$$

where N_0_ is the mean TCID_50_ of the positive (virus) control group, and Nx is the mean TCID_50_ of the test (disinfection) group. The method systematically sets a minimum value of log Nx as ≤ 0.5 if no virus multiplication is observed in the highest concentration. And the corresponding killing log-value is denoted with the sign “≥”.

## Results

### Neutralizer identification test

As shown in Table 2, the results of the neutralizer identification test for chlorinated disinfectants showed that 1 g/L sodium thiosulfate and 0.5 g/L sodium thiosulfate dissolved in DMEM maintenance solution were effective in terminating the residual toxicity of disinfectants containing 1000 mg/L of effective chlorine and 500 mg/L of effective chlorine, respectively, and that the neutralizer and neutralization products had no adverse effects on the cells and virus. Therefore, they were selected as neutralizing agents for the SARS-CoV-2 inactivation test with chlorinated disinfectants.

**Table 2:** Neutralizer identification test results.

Group^a^	TCID_50_ log10							
	ChlorineTest I (1000 mg/L)	ChlorineTest II (500 mg/L)	DNBTest I (850 mg/L)	DNBTest II (170 mg/L)	DNCTest I (850 mg/L)	DNCTest II (170 mg/L)	EthanolTest I (75%)	EthanolTest II (20%)
D + V	0.50 ± 0.00^b^	0.50 ± 0.00	0.50 ± 0.00	1.83 ± 0.12	0.50 ± 0.00	0.50 ± 0.00	0.50 ± 0.00	3.92 ± 0.12
D + V + N	0.50 ± 0.00	1.33 ± 0.12	0.50 ± 0.00	3.25 ± 0.20	0.50 ± 0.00	2.92 ± 0.12	0.50 ± 0.00	4.17 ± 0.12
N + V	5.17 ± 0.12^c^	5.08 ± 0.12	5.17 ± 0.12	5.17 ± 0.12	5.08 ± 0.12	5.08 ± 0.12	5.17 ± 0.12	5.17 ± 0.12
D + N + V	5.08 ± 0.12	5.17 ± 0.12	5.00 ± 0.00	5.08 ± 0.12	5.08 ± 0.12	5.08 ± 0.12	5.00 ± 0.00	5.08 ± 0.12
V	5.42 ± 0.12	5.33 ± 0.12	5.33 ± 0.12	5.42 ± 0.12	5.17 ± 0.12	5.17 ± 0.12	5.17 ± 0.12	5.17 ± 0.12

^a^*D* disinfectant, *V* viral suspension, *N* neutralizer.

^b^If the value is equal to 0.5, we did not find a live virus after the treatment.

^c^If this value is bigger than 5, which means the virus is not affected. The small variation in the values is due to small changes in the virus titers used in different experiments.

QAC (DNB and DNC) disinfectant neutralizer studies showed that 0.5 g/L sodium thiosulfate + 0.5 g/L lecithin and 0.1 g/L sodium thiosulfate + 0.1 g/L lecithin dissolved in DMEM maintenance solution effectively discontinued the residual toxicity of the double-chain quaternary ammonium salts disinfectants at 850 mg/L and 185 mg/L dilutions, respectively, and that neither the neutralizer nor neutralization product had adverse effects on cells and viruses. Therefore, they were selected as neutralizing agents for the double-chain quaternary ammonium salt disinfectants against the SARS-CoV-2 inactivation experiment.

The results of the ethanol neutralizer study showed that DMEM maintenance solution was effective in suspending 75% ethanol and 20% ethanol residual toxicity and that the neutralizer and neutralization products had no adverse effects on the cells and viruses. It was identified as a neutralizer for the ethanol disinfectant against the SARS-CoV-2 inactivation experiment.

### Effect of chlorinated disinfectants

As shown in Table 3, our results showed that during the exposure interval set in this study, it took 20 min for the disinfectant containing 250 mg/L of available chlorine to efficiently inactivate SARS-CoV-2 (≥ 4.75 Log_10_ reductions). The disinfectant containing 500 mg/L of available chlorine needed no more than 5 min to efficiently inactivate SARS-CoV-2, and the disinfectant containing 1000 mg/L of available chlorine needed less than 0.5 min to efficiently inactivate the virus.

**Table 3:** Inactivation effects of chlorinated disinfectants on SARS-CoV-2.

Concentration of available chlorine (mg/L)	Log_10_ reduction in infectious SARS-CoV-2 titer achieved Mean (Min–Max)			
	0.5 min	5 min	10 min	20 min
250	–	3.25 (3.00–3.50)	4.00 (3.75–4.25)	≥ 4.75^a^
500	3.58 (3.50–3.75)	≥ 4.75	≥ 4.75	≥ 4.75
1000	≥ 4.75	≥ 4.75	≥ 4.75	≥ 4.75

^a^The value of ≥ 4.75 means that no live virus is observed after the treatment, while the smaller values mean that a different amount of virus survives after the treatment. Results shown are the average of at least three replicate experiments.

### Effect of QAC

As shown in Table 4, it is evident that even a 1000-fold dilution (170 mg/L) of DNB disinfectant can efficiently inactivate SARS-CoV-2 after 5 min of action time. At a DNB concentration of 212 mg/L (1:800 dilution), the virus was inactivated with similar efficacy to the 1:1000 dilution; 283 mg/L (1:600) and higher concentrations of DNB required only 0.5 min to inactivate the virus efficiently. The results of further tests using standard chemically pure DNC are shown in Table 5 and were similar to those of DNB.

**Table 4:** Effect of Di-N-decyl dimethyl ammonium bromide (DNB) on SARS-CoV-2 inactivation.

Dilution ratio (concentration, mg/L)	Log_10_ reduction in infectious SARS-CoV-2 titer achieved Mean (Min–Max)			
	0.5 min	1 min	5 min	10 min
1:1000 (170)	2.50 (2.25–2.75)	≥ 4.92^a^	≥ 4.92	≥ 4.92
1:800 (212)	3.59 (3.50–3.75)	≥ 4.92	≥ 4.92	≥ 4.92
1:600 (283)	≥ 4.92	≥ 4.92	≥ 4.92	≥ 4.92
1:400 (425)	≥ 4.92	≥ 4.92	≥ 4.92	≥ 4.92
1:200 (850)	≥ 4.92	≥ 4.92	≥ 4.92	≥ 4.92

^a^The value of ≥ 4.92 means that no live virus is observed after the treatment, while the smaller values indicate that a different amount of virus survives after the treatment.

**Table 5:** Effect of Di-N-decyl dimethyl ammonium chloride (DNC) on SARS-CoV-2 inactivation.

Concentration (mg/L)	Log_10_ reduction in infectious SARS-CoV-2 titer achieved Mean (Min ~ Max)			
	0.5 min	1 min	5 min	10 min
170	2.50 (2.25*2.75)	≥ 4.92^a^	≥ 4.92	≥ 4.92
212	3.59 (3.50–3.75)	≥ 4.92	≥ 4.92	≥ 4.92
283	≥ 4.92	≥ 4.92	≥ 4.92	≥ 4.92
425	≥ 4.92	≥ 4.92	≥ 4.92	≥ 4.92
850	≥ 4.92	≥ 4.92	≥ 4.92	≥ 4.92

^a^The value of ≥ 4.92 means that no live virus is observed after the treatment, while the smaller values indicate that a different amount of virus survives after the treatment.

### Effect of ethanol

As shown in Table 6, 20% of ethanol was unable to inactivate the virus, while 30% of ethanol could inactivate the virus efficiently within 1 min. A disinfectant solution containing 40% or more ethanol required only 0.5 min to inactivate SARS-CoV-2 efficiently. All concentrations above 30% provided at least a 4 log 10 reduction in viral titers within a 0.5 min contact time.

**Table 6:** Effect of ethanol on SARS-CoV-2 inactivation.

Concentration (%)	Log_10_ reduction in infectious SARS-CoV-2 titer achieved Mean (Min ~ Max)			
	0.5 min	1 min	3 min	5 min
20	1.08 (0.75–1.50)	1.33 (1.00–1.50)	1.75 (1.50–2.00)	1.92 (1.50–2.25)
30	4.42 (4.25–4.50)	≥ 4.75^a^	≥ 4.75	≥ 4.75
40	≥ 4.75	≥ 4.75	≥ 4.75	≥ 4.75
50	≥ 4.75	≥ 4.75	≥ 4.75	≥ 4.75
60	≥ 4.75	≥ 4.75	≥ 4.75	≥ 4.75
75	≥ 4.75	≥ 4.75	≥ 4.75	≥ 4.75

^a^The value of ≥ 4.75 means that no live virus is observed after the treatment, while the smaller values indicate that a different virus survives after the treatment.

### Effects of heat

As shown in Table 7, we tested the effect of different temperatures on the inactivation of SARS-CoV-2, and the results showed that the virus could be efficiently inactivated following exposure to a temperature of 56 °C for 30 min. Besides, we found that exposure to temperatures of 70 °C and 90 °C could inactivate the virus in 10 min and 5 min, respectively.

**Table 7:** Effects of heat on SARS-CoV-2 inactivation.

Temperature (°C)	Log_10_ reduction in infectious SARS-CoV-2 titer achieved Mean (Min–Max)			
	5 min	10 min	20 min	30 min
56	_	4.25 (4.00–4.50)	4.84 (4.75–5.00)	≥ 5.17^a^
70	–	≥ 5.17	≥ 5.17	–
90	≥ 5.17	–	–	–

^a^The value of ≥ 5.17 means that no live virus is observed after the treatment, while the smaller values indicate that a different amount of virus survives after the treatment.

## Discussions

Limited laboratory data are available on the efficacy of the various disinfectants of SARS-CoV-2. This study, which examined the laboratory inactivation efficacy of chlorinated disinfectants, QAC, ethanol, and heat against SARS-CoV-2, is the first to confirm that QAC showed an appreciable effect on this virus. The results provide important disinfection information for SARS-CoV-2 that can be applied globally. Currently, the COVID-19 pandemic is ongoing, but practical experience from China indicates that stringent disinfection and control measures were effective in limiting person-to-person transmission.

Chlorinated disinfectants are high-efficacy disinfectants. Even low concentrations of chlorine and sodium hypochlorite achieve rapid activity against viral nucleic acid^17^. However, whether chlorine acts preferably against the viral genome rather than against the viral capsid remains unknown^18^. WHO recommends that regular household disinfectants containing 0.1% sodium hypochlorite (1000 mg/L) should be applied to various household surfaces^19^. Previous studies have reported that MERS-CoV or endemic human coronaviruses (HCoV) can persist on inanimate surfaces, such as metal, glass, or plastic, for up to 9 days, but can be efficiently inactivated by surface disinfection procedures with 0.1% sodium hypochlorite within 1 min^20^. Our test results confirmed that this method was also effective against SARS-CoV-2, which effectively inactivated the virus in less than 0.5 min at this concentration. This result was also consistent with previous reports of complete disruption of the SARS-CoV genome using 0.1% sodium hypochlorite (1000 mg/L) for 1 min^21^. As these previous experiments were not designed for a shorter duration of action^21^, our results have substantiated the efficacy of a shorter duration of action. This result also demonstrated that the SARS-CoV-2 virus was indeed similar to SARS-CoV in its resistance to chlorinated disinfectants. The US CDC recommends using 1/3 cup of bleach added to 1 gallon of water for surfaces exposed to COVID-19 patients, which is approximately 64 times diluted and has an available chlorine content of roughly 781 mg/L. According to the results of the present study, this disinfectant could efficiently inactivate SARS-CoV-2 within 5 min. It is important to note that chlorinated disinfectants are only used on environmental surfaces and are not to be ingested into the human body, as ingestion of bleach is known to produce esophageal stenosis^22^, hypernatraemia and hyperchloraemic acidosis^23^. Hypochlorous acid occurs when chlorinated disinfectants are dissolved in water^24^. Hypochlorous acid can be absorbed, ingested, or absorbed percutaneously into humans or other animals and is irritating to skin and mucous membranes^25^. Excess chlorinated disinfectants take some time to degrade when they enter the natural environment, especially when they reach wild plants and animals^26^. Chlorine may exhibit acute toxicity to aquatic organisms at concentrations less than or equal to 1 mg/L^27^.

QAC disinfectants are a class of low efficacy disinfectants with stable and low corrosive properties, mainly used for the disinfection of hands, skin, mucous membranes, and the surface of environmental objects. Benzalkonium chloride is a representative quaternary ammonium salt disinfectant and is commonly used with ethanol formulations. However, the killing effect of benzalkonium chloride on SARS-CoV-2 has been controversial^28^. The US CDC did not endorse any benzalkonium chloride-based hand sanitizer against COVID-19 because the supporting research is neither current nor uniformly asserted. Kampf G et al.^20^ reported that 0.05–0.2% benzalkonium chloride or 0.02% chlorhexidine digluconate was less effective for MERS-CoV or other previous HCoV. The benzalkonium chloride-based product (Dettol Hospital Concentrate), which was active against the non-enveloped human coxsackievirus, was ineffective in inactivating human coronavirus (enveloped) and non-enveloped viruses^29^.

DNB and DNC are also well-established QAC disinfectant commodities that were examined in previous investigations against SARS-CoV. The SARS-CoV was inactivated by DNC (5000 mg/L, 30 min) with a smaller reduction factor of 3.25 log10, regardless of the type of organic load^10^. Our study unexpectedly found that commercialized DNB was highly effective in inactivating SARS-CoV-2 at very low concentrations, inactivating the virus in 0.5 min after exposure to a 600-fold dilution (283 mg/L), and efficiently inactivating the virus within minutes, even at a 1000-fold dilution (170 mg/L). To confirm this result, we repeated the DNB experiment with pure DNC, and the results were consistent (Table 5). A possible reason for this is that the quaternary ammonium compounds are markedly more specific in their antimicrobial mechanism. Even very low concentrations cause damage to the cytoplasmic membrane due to the perturbation of the bilayers by the molecular alkyl chains^30^. The results of this study also supported the recommendation of some QAC by the US EPA as candidate commodities that could kill SARS-COV-2^14^. As quaternary salt disinfectants are less corrosive than chlorinated disinfectants, they can be used for the internal disinfection of transportation equipment, such as aircraft and high-speed trains. The results of this study showed that DNB could effectively inactivate SARS-CoV-2 in a very short time at a dilution ratio of 1:200 as recommended by the manufacturer. The present results support reducing the dose of QAC disinfectants, which helps to reduce the environmental load of QAC. After all, elevated QAC exposure might jolt the spread of antibiotic resistance and connect with other environmental issues^31,32^. QACs are absorbed in the environment faster than they are degraded, which results in their accumulation in the environment, where they are toxic to aquatic and terrestrial organisms^33^. It is also worth noting that exposure to QAC may trigger asthma in humans^34^ or increase pulmonary cell damage and inflammation in rats^35^. QACs also have potential mutagenicity and reproductive toxicity^36^. Some QACs are mutagenic and could damage animal DNA and human lymphocyte DNA at much lower levels (as low as 0.3 mg/L)^37^.

Ethanol disinfectant is an intermediate efficacy disinfectant, mainly used for hand, environmental surface, and medical equipment surface disinfection. A previous study suggests that the viral envelope might be a major target site of ethanol^18^. Guidelines and standards recommend common concentrations of 70–75%. In previous studies, 80% and 85% of ethanol concentrations have also been reported to kill SARS-CoV within 0.5 min^10^. For SARS-CoV-2, the WHO recommends the use of hand rub formulations and alcohols. Original WHO formulation I consists of 80% (vol/vol) ethanol, 1.45% (vol/vol) glycerol, and 0.125% (vol/vol) hydrogen peroxide. Recent studies have demonstrated that SARS-CoV-2 is highly susceptible to both the original and modified WHO formulations. Both formulations were able to reduce viral titers to the background level within 0.5 min^13^. This was consistent with the results of our study. Additionally, it was found that reducing the concentration of ethanol did not affect its efficacy against SARS-CoV-2. This may provide some help for people with alcohol allergies; nonetheless, these people can also adopt WHO formulation II, which consists of 75% (vol/vol) 2-propanol, 1.45% (vol/vol) glycerol, and 0.125% (vol/vol) hydrogen peroxide^13^. MERS-CoV or other HCoV can be efficiently inactivated by surface disinfection procedures with 62–71% ethanol^20^. The results of the present study further suggest that SARS-CoV-2 has a similar resistance to ethanol as several previous coronaviruses. Ethanol, at a concentration of 60–75%, can be applied for routine disinfection, and low concentrations can be combined with other ingredients to enhance its effectiveness and safety. Given the volatile nature of alcohol, only a moderate increase in its concentration is needed to ensure its effectiveness.

Heat disinfection is the most common means of physically killing the virus. The virus’s exposure to a temperature of 56 °C over 30 min reduced the viral titer to an undetectable level, except if SARS-CoV is associated with proteins, such as 20% fetal calf serum, which conveys protection to the virus. In this case, the temperature needs to reach 60 °C over 30 min to bring the viral titer below the detection limit^11^. Another study confirmed that SARS-CoV lost its infectivity after 90, 60, and 30 min exposure to temperatures of 56 °C, 67 °C, and 75 °C, respectively^15^. Another Chinese study found that exposure to 56 °C for 30 min, or 70 °C for 15 min, could inactivate SARS-CoV^38^. The present study results are consistent with previous investigations in which exposure to a temperature of 56 °C for 30 min effectively inactivated SARS-CoV-2^38–40^. This further suggests that SARS-CoV-2 has similar heat resistance to SARS-CoV.

To the best of our knowledge, this study is the first to use the SARS-CoV-2 strain for a comprehensive comparative evaluation of the effect of multiple disinfectants on virus inactivation^41^. For each disinfectant or method, we set up concentration and contact time stratification, identified neutralizers for the chemical disinfectants, and tested and compared the laboratory’s disinfection effects of thermal and low, medium, and high-efficiency disinfectants against SARS-CoV-2. Furthermore, to stimulate protein protection, the protocol of the test included 3% bovine serum albumin. As viral resistance is enhanced under protective conditions containing higher organic matter, especially protein-containing organic matter, 3% albumin concentration conditions are moderate; 0.3% has been studied in alcohol disinfectant studies^13^, and 20% was used in a heat inactivation study^11^, the latter increasing the resistance time of SARS-CoV to a temperature of 56 °C.

The limitation of the current research is that the study ended with viral inactivation and did not simultaneously focus on destroying the viral genome. A previous study using 500 mg/L chlorine to treat SARC-Cov for 30 min showed that the genome of the virus was damaged^21^, which indicates that chlorine could damage the viral genome. Next, we will study the dose needed for the complete inactivation of SARC-Cov-2. Second, the use of the virus suspension test method necessitated the guarantee of non-toxicity to cells, which limited the evaluation of the disinfection effect of substances such as chlorhexidine. Moreover, the types of disinfectants examined were only a few common varieties; however, this helps the results of this study guide disinfectants’ practical application. Our study did not include some of the more common household disinfectants, such as soap, because there are too many varieties among soaps to be easily standardized in the laboratory. A study showed that soaps contain surfactants that dissolve the lipid-bilayer, an integral part of the SARS-CoV-2 envelope, leading to the virus’s deactivation^42^.

In summary, in this study, we identified four chemical disinfectant neutralizers using the suspension quantification test method and confirmed that chlorinated disinfectants, QAC (DNB and DNC), ethanol, and heat were effective in inactivating SARS-CoV-2. This study has demonstrated that SARS-CoV-2 is similar to SARS-CoV in its resistance to disinfectants. Additionally, the QAC disinfectants, DNB and DNC, exhibited high efficiency with low dose effectiveness and short reaction times and should play a more significant role in the global fight against COVID-19. However, we should still consider the possible impact of chlorinated disinfectants and QACs on the ecological environment, and large discharges of these disinfectants into the environment should be limited to avoid toxicity to aquatic organisms.
